# Supplementary material for: Nascent MSKIK peptide cancels ribosomal stalling by arrest peptides in Escherichia coli
Source: J Biol Chem. 2023 Apr 5;299(5):104676. doi: 10.1016/j.jbc.2023.104676 (PMC10192921; doi:10.1016/j.jbc.2023.104676)
Supplement: Supporting information [file mmc1.docx]

Supporting information

**Nascent MSKIK Peptide Cancels Ribosomal Stalling by Arrest Peptides in *Escherichia coli***

Teruyo Ojima-Kato,* Yuma Nishikawa, Yuki Furukawa, Takaaki Kojima, Hideo Nakano

Ojima-Kato et al., Figure S1

**Figure S1. Comparison of protein production by dot blot and SDS-PAGE analysis**

This data supports Figure 1 in the main manuscript. The numbers correspond to those of clones in Table 1. M: size marker.

(A) Three-fold diluted cell lysate samples and non-diluted no-tagged samples were visualized with TMB color development. The triplicated results are shown.

(B) CBB staining images of cell lysates of 36 variants and no-tagged scFvs. The arrows indicate the target protein band size.

Ojima-Kato et al., Figure S2

**Figure S2. SDS-PAGE of auto-induction samples**

CBB-stained SDS-PAGE gels for expression of scFv in *E. coli* Shuffle T7 Express cells by autoinduction at 30˚C for 72 h. M: size marker. The numbers correspond to the codon patterns listed in Table 1. The arrows indicate the band representing the protein of interest.

Ojima-Kato et al., Figure S3

**Figure S3. Fluorescence detection of SDS-PAGE gels of cell-free protein synthesis products**

Cell-free protein synthesis products incorporating fLys t-RNA with the 36 SKIK codon variants were analyzed by fluorescence detection using a Typhoon FLA9000. M: size marker; NC: negative control. The bands indicated by the arrows are the protein of interest.

Ojima-Kato et al., Figure S4

**Figure S4. The minimum free energies and optimal secondary structures of the mRNAs of the 36 SKIK variants and no-tagged mRNA**

The values and structures were predicted by RNAfold. Whole represents the entire mRNA from the transcription starting point to the T7 terminator. Short represents a focused region consisting of 64 bases that contains the Shine-Dalgano, start codon, and SKIK-encoding sequences.

(A) Minimum free energy of the centroid secondary structure in dot-bracket notation is shown for each variant. Clone numbers correspond to the SKIK-encoding codon variants listed in Table 1. The left and right y-axes show the calculated energy values for whole and short constructs, respectively.

(B) The centroid secondary structures presented correspond to SKIK-tagged clone No. 1 and No tag.

Ojima-Kato et al., Figure S5

**Figure S5. The entire gel and membrane images corresponding to Figure 2.**

Rabbit heavy chain without Fc region (Hc) and light chain (Lc). Plus and minus indicate the presence and absence of the SKIK tag, respectively. M represents size marker.

(A) The agarose gel image.

(B) The nitrocellulose membrane image of Western blotting.

Ojima-Kato et al., Figure S6

**Figure S6. Protein production in *E. coli* with N-terminal SKX, KKX, and AKX tags**

CBB staining of SDS-PAGE gels is shown. Arrows indicate bands representing the target product. M: size marker.

Table S1. DNA Primers used in this study

| Name | Sequence (5'→3') | Discription |
| --- | --- | --- |
| SKIK1111F | ATGTCTAAAATTAAAGACCCTATGCTGACC |  |
| SKIK1111R | GGTCTTTGATTTTAGACATATGTATATCTC |  |
| SKIK1121F | ATGTCTAAAATCAAAGACCCTATGCTGACC |  |
| SKIK1121R | GGTCTTTGATTTTAGACATATGTATATCTC |  |
| SKIK1131F | ATGTCTAAAATAAAAGACCCTATGCTGACC |  |
| SKIK1131R | GGTCTTTGATTTTAGACATATGTATATCTC |  |
| SKIK1211F | ATGTCTAAGATTAAAGACCCTATGCTGACC |  |
| SKIK1211R | GGTCTTTAATTTTAGACATATGTATATCTC |  |
| SKIK1221F | ATGTCTAAGATCAAAGACCCTATGCTGACC |  |
| SKIK1221R | GGTCTTTGATCTTAGACATATGTATATCTC |  |
| SKIK1231F | ATGTCTAAGATAAAAGACCCTATGCTGACC |  |
| SKIK1231R | GGTCTTTTATCTTAGACATATGTATATCTC |  |
| SKIK2111F | ATGTCCAAAATCTAAAGACCCTATGCTGACC |  |
| SKIK2111R | GGTCTTTAATTTTGGACATATGTATATCTC |  |
| SKIK2121F | ATGTCCAAAATCAAAGACCCTATGCTGACC |  |
| SKIK2121R | GGTCTTTGATTTTGGACATATGTATATCTC |  |
| SKIK2131F | ATGTCCAAAATAAAAGACCCTATGCTGACC |  |
| SKIK2131R | GGTCTTTTATTTTGGACATATGTATATCTC |  |
| SKIK2211F | ATGTCCAAGATTAAAGACCCTATGCTGACC |  |
| SKIK2211R | GGTCTTTAATCTTGGACATATGTATATCTC |  |
| SKIK2221F | ATGTCCAAGATCAAAGACCCTATGCTGACC |  |
| SKIK2221R | GGTCTTTGATCTTGGACATATGTATATCTC |  |
| SKIK2231F | ATGTCCAAGATAAAAGACCCTATGCTGACC |  |
| SKIK2231R | GGTCTTTGATCTTGGACATATGTATATCTC |  |
| SKIK3111F | ATGTCAAAAATTAAAGACCCTATGCTGACC |  |
| SKIK3111R | GGTCTTTAATTTTTGACATATGTATATCTC |  |
| SKIK3121F | ATGTCAAAAATCAAAGACCCTATGCTGACC |  |
| SKIK3121R | GGTCTTTGATTTTTGACATATGTATATCTC |  |
| SKIK3131F | ATGTCAAAAATAAAAGACCCTATGCTGACC |  |
| SKIK3131R | GGTCTTTTATTTTAGACATATGTATATCTC |  |
| SKIK3211F | ATGTCAAAGATTAAAGACCCTATGCTGACC |  |
| SKIK3211R | GGTCTTTAATCTTTGACATATGTATATCTC |  |
| SKIK3221F | ATGTCAAAGATCAAAGACCCTATGCTGACC |  |
| SKIK3221R | GGTCTTTAATCTTTGACATATGTATATCTC |  |
| SKIK3231F | ATGTCAAAGATAAAAGACCCTATGCTGACC |  |
| SKIK3231R | GGTCTTTTATCTTTGACATATGTATATCTC |  |
| SKIK4111F | ATGTCGAAAATTAAAGACCCTATGCTGACC |  |
| SKIK4111R | GGTCTTTAATTTTCGACATATGTATATCTC |  |
| SKIK4121F | ATGTCGAAAATCAAAGACCCTATGCTGACC |  |
| SKIK4121R | GGTCTTTGATTTTCGACATATGTATATCTC |  |
| SKIK4131F | ATGTCGAAAATAAAAGACCCTATGCTGACC |  |
| SKIK4131R | GGTCTTTTATTTTCGACATATGTATATCTC |  |
| SKIK4211F | ATGTCGAAGATTAAAGACCCTATGCTGACC |  |
| SKIK4211R | GGTCTTTAATCTTCGACATATGTATATCTC |  |
| SKIK4221F | ATGTCGAAGATCAAAGACCCTATGCTGACC |  |
| SKIK4221R | GGTCTTTGATCTTCGACATATGTATATCTC |  |
| SKIK4231F | ATGTCGAAGATAAAAGACCCTATGCTGACC |  |
| SKIK4231R | GGTCTTTTATCTTCGACATATGTATATCTC |  |
| SKIK5111F | ATGAGTAAAATTAAAGACCCTATGCTGACC |  |
| SKIK5111R | GGTCTTTAATTTTACTCATATGTATATCTC |  |
| SKIK5121F | ATGAGTAAAATCAAAGACCCTATGCTGACC |  |
| SKIK5121R | GGTCTTTGATTTTACTCATATGTATATCTC |  |
| SKIK5131F | ATGAGTAAAATAAAAGACCCTATGCTGACC |  |
| SKIK5131R | GGTCTTTTATTTTACTCATATGTATATCTC |  |
| SKIK5211F | ATGAGTAAGATTAAAGACCCTATGCTGACC |  |
| SKIK5211R | GGTCTTTAATCTTACTCATATGTATATCTC |  |
| SKIK5221F | ATGAGTAAGATCAAAGACCCTATGCTGACC |  |
| SKIK5221R | GGTCTTTGATCTTACTCATATGTATATCTC |  |
| SKIK5231F | ATGAGTAAGATAAAAGACCCTATGCTGACC |  |
| SKIK5231R | GGTCTTTTATCTTACTCATATGTATATCTC |  |
| SKIK6111F | ATGAGCAAAATTAAAGACCCTATGCTGACC |  |
| SKIK6111R | GGTCTTTAATTTTGCTCATATGTATATCTC |  |
| SKIK6121F | ATGAGCAAAATCAAAGACCCTATGCTGACC |  |
| SKIK6121R | GGTCTTTGATTTTGCTCATATGTATATCTC |  |
| SKIK6131F | ATGAGCAAAATAAAAGACCCTATGCTGACC |  |
| SKIK6131R | GGTCTTTTATTTTGCTCATATGTATATCTC |  |
| SKIK6211F | ATGAGCAAGATTAAAGACCCTATGCTGACC |  |
| SKIK6211R | GGTCTTTAATCTTGCTCATATGTATATCTC |  |
| SKIK6221F | ATGAGCAAGATCAAAGACCCTATGCTGACC |  |
| SKIK6221R | GGTCTTTGATCTTGCTCATATGTATATCTC |  |
| SKIK6231F | ATGAGCAAGATAAAAGACCCTATGCTGACC |  |
| SKIK6231R | GGTCTTTTATCTTGCTCATATGTATATCTC |  |
| r1scFv_SKG-F | ATGTCTAAAGGTGACCCTATGCTGACC |  |
| r1scFv_SKG-R | GGTCACCTTTAGACATATGTATATCTC |  |
| r1scFv_SKL-F | ATGTCTAAATTAGACCCTATGCTGACC |  |
| r1scFv_SKL-R | GGTCTAATTTAGACATATGTATATCTC |  |
| r1scFv_SKH-F | ATGTCTAAACATGACCCTATGCTGACC |  |
| r1scFv_SKH-R | GGTCATGTTTAGACATATGTATATCTC |  |
| r1scFv_SKY-F | ATGTCTAAATATGACCCTATGCTGACC |  |
| r1scFv_SKY-R | GGTCATATTTAGACATATGTATATCTC |  |
| r1scFv_SKE-F | ATGTCTAAAGAAGACCCTATGCTGACC |  |
| r1scFv_SKE-R | GGTCTTCTTTAGACATATGTATATCTC |  |
| r1scFv_SKF-F | ATGTCTAAATTTGACCCTATGCTGACC |  |
| r1scFv_SKF-R | GGTCAAATTTAGACATATGTATATCTC |  |
| r1scFv_KKG-F | ATGAAAAAAGGTGACCCTATGCTGACC |  |
| r1scFv_KKG-R | GGTCACCTTTTTTCATATGTATATCTC |  |
| r1scFv_KKL-F | ATGAAAAAATTAGACCCTATGCTGACC |  |
| r1scFv_KKL-R | GGTCTAATTTTTTCATATGTATATCTC |  |
| r1scFv_KKH-F | ATGAAAAAACATGACCCTATGCTGACC |  |
| r1scFv_KKH-R | GGTCATGTTTTTTCATATGTATATCTC |  |
| r1scFv_KKY-F | ATGAAAAAATATGACCCTATGCTGACC |  |
| r1scFv_KKY-R | GGTCATATTTTTTCATATGTATATCTC |  |
| r1scFv_KKE-F | ATGAAAAAAGAAGACCCTATGCTGACC |  |
| r1scFv_KKE-R | GGTCTTCTTTTTTCATATGTATATCTC |  |
| r1scFv_KKF-F | ATGAAAAAATTTGACCCTATGCTGACC |  |
| r1scFv_KKF-R | GGTCAAATTTTTTCATATGTATATCTC |  |
| r1scFv_AKG-F | ATGGCAAAAGGTGACCCTATGCTGACC |  |
| r1scFv_AKG-R | GGTCACCTTTTGCCATATGTATATCTC |  |
| r1scFv_AKL-F | ATGGCAAAATTAGACCCTATGCTGACC |  |
| r1scFv_AKL-R | GGTCTAATTTTGCCATATGTATATCTC |  |
| r1scFv_AKH-F | ATGGCAAAACATGACCCTATGCTGACC |  |
| r1scFv_AKH-R | GGTCATGTTTTGCCATATGTATATCTC |  |
| r1scFv_AKY-F | ATGGCAAAATATGACCCTATGCTGACC |  |
| r1scFv_AKY-R | GGTCATATTTTGCCATATGTATATCTC |  |
| r1scFv_AKE-F | ATGGCAAAAGAAGACCCTATGCTGACC |  |
| r1scFv_AKE-R | GGTCTTCTTTTGCCATATGTATATCTC |  |
| r1scFv_AKF-F | ATGGCAAAATTTGACCCTATGCTGACC |  |
| r1scFv_AKF-R | GGTCAAATTTTGCCATATGTATATCTC |  |
| F1 | ATCTCGATCCCGCGAAATTAATACG |  |
| R1 | TCCGGATATAGTTCCTCCTTTCAG |  |
| SecM-F | GCCGAACCAAACGCGCCCGCAAAAG | SecM cloning |
| SecM-R | GGTGAGGCGTTGAGGGCCAGCAC | SecM cloning |
| CmlA_sfGFP-F | AAGAATGCGATGCAAGTAAAGGTGAAGAACTGTTTAC | insertion of CmlA leader and SKIK-CmlA leader |
| CmlA_sfGFP-R | ATCGCATTCTTCATATGGATATCTCCTTCTTAAAG | insertion of CmlA leader |
| SKIK-CmlA_sfGFP-R | ATCGCATTCTTTTTTATTTTAGACATATGGATATCTCCTTCTTAAAG | insertion of SKIK-CmlA leader |
| WPPP_sfGFP-F | CGGGATTTGGCCGCCCCCTGCAAGTAAAGGTGAAGAAC | insertion of WPPP and SKIK-WPPP |
| WPPP_sfGFP-R | CCAAATCCCGTACTTCTGGAACATATGGATATCTCCTTCTTAAAG | insertion of WPPP AP |
| SKIK-WPPP_sfGFP-R | CCAAATCCCGTACTTCTGGAATTTTATTTTAGACATATGGATATCTCCTTC | insertion of SKIK-WPPP |
| His6-TAA-F | CATCACCATCACCATCATTAAAGATCCGGC | linearization of pET22b-AP–secM(-AP) and pET22b-SKIK-AP–secM(-AP) |
| SecMAP-R | TGGTTCGGCAGGGCCAGCACGGATGCC | linearization of pET22b-AP–secM(-AP) and pET22b-SKIK-AP–secM(-AP) |
| sfGFP-F | GCTGGCCCTGCCGAACCAGCAAGTAAAGGTGAAGAACTG | insertion of sfGFP into linearized vector |
| sfGFP-R | CTTTAGTGGTGGTGGTGGTGGTGCAGTTTATACAGTTCATCCATGC | insertion of sfGFP into linearized vector |

Table S2. Plasmid DNA sequences used in this study

| Gene | DNA sequence | Discription |
| --- | --- | --- |
| r1scFv-His tag | ATGGACCCTATGCTGACCCAGACTCCAGCCTCCGTGTCTGCAGCTGTGGGAGGCACAGTCACCATCAAGTGCCAGGCCAGTGAGAACATTTACACCTCTTTAGCCTGGTATCAGCAGAAACCAGGGCACTCTCCTAAGCTCCTGATCTATTCTGCATCCACTCTGGCATCTGGGGTCGCATCGCGGTTCAAAGGCAGTGGATCTGGGACACAGTTCACTCTCACCATCAGCGGCGTGCAGTGTGATGATGCTGCCACTTACTATTGTCAATGTAGTGCTTATGGTAGGAGTGGTAATTCTTTCGGCGGAGGGACCGAGGTGGTGGTCAACGGTGATCCAGTTGCACCTACTGGAGGTGGTGGATCCGGCGGTGGCGGTTCTGGTGGAGGTGGATCTCAGTCGCTGGAGGAGTCCGGGGGTCGCCTGGTCACGCCTGGGACACCCCTGACACTCACCTGCACAGTCTCTGGATTCTCCCTCAGTAGTTATGCAATGAGCTGGGTCCGCCAGGCTCCAGGGAAGGGGCTGGAATGGATCGGAAGTATTGGTACTGGTGGTAGCACATACTACGCGATCTGGGCGAAAGGCCGATTCACCATCTCCAAAACCTCGACCACGGTGGGTCTGAAAATCATCAGTCCGACAACCGAGGACACGGCCACCTATTTCTGTGCCCAGAATAGTTATGGTTATGTTGGTGTTAGGGAATATTTTAAGTTGTGGGGCCCAGGCACCCTGGTCACCGTCGGCGGTCATCATCATCACCATCACTAA | Basic sequence without SKIK tag is shown. |
| HcG_22 with/without SKIK | ATG[TCTAAAATAAAA]CAGTCGTTGGAGGAGTCCGGGGGAGGCCTGGTCCAGCCTGAGGGATCCCTGGCACTCACCTGCAAAGCCTCTGGATTCACCATCAGTAGCAGCTACTACATGTGCTGGGTCCGCCAGGCTCCAGGGAAGGGGCTGGAGTGGATCGGATGCATTTATGCTGGTAGTGGTGGTACATACTACGCGAGCTGGGCGAAAGGCCGATTCACCATCTCCAAGTCCTCGTCGACCACGGTGACTCTGCAAATGACCAGTCTGACAGCCGCGGACACGGCCACCTATTTCTGTGCGAGGGACGTTGATGTTAGTGGTTATGGTCTGGACTTGTGGGGCCCAGGCACCCTGGTCACCGTCTCCTCAGGGCAACCTAAGGCTCCATCAGTCTTCCCACTGGCACCGTGTTGTGGTGATACCCCGAGCAGCACCGTTACCCTGGGTTGTCTGGTTAAAGGTTATCTGCCGGAACCGGTTACCGTTACCTGGAATAGCGGCACCCTGACCAATGGTGTTCGTACCTTTCCGAGCGTTCGTCAGAGCAGCGGTCTGTATAGCCTGAGCAGCGTTGTTAGCGTTACCAGCAGCAGCCAGCCGGTTACCTGTAATGTTGCACATCCGGCTACCAATACCAAAGTTGATAAAACCGTTGCACCGAGCACCTGTGGCGGTGGTGGGAGCGCCCAGCTCGAAAAGGAGCTGCAAGCCCTGGAGAAGGAGAACGCCCAGCTCGAATGGGAGCTCCAGGCCCTGGAGAAGGAGCTGGCCCAGAAGGGCGGTACCATGTACCCATACGATGTTCCAGATTACGCTTAATAA | VH-CH1 region of rabbit antibody (BAX56587) with leucine zipper and HA tag. |
| Lc_22 with/without SKIK | ATG[TCTAAAATAAAA]GATGTTGTGATGACCCAGACTCCAGACTCCGTGTCTGCAGCTGTGGGAGGCACAGTCACCATCAATTGCCAGGCCAGTGAGAGCATTTATAGCAATTTAGCCTGGTATCAGCAGAAACCAGGGCAGCCTCCCAAGCTCCTGATCTATGCTGCATCGAAACTGGCATCTGGGGTCCCATCGCGGTTCAAAGGCAGTGGATCTGGGACACAGTTCACTCTCACCATCAGCGACCTGGAGTGTGCCGATGCTGCCACTTACTACTGTCAATGTACTTATTATGGTAGTAGTGCTGTTCCTAATGCTTTCGGCGGAGGGACCGAGGTGGTGGTCAAAGGTGATCCAGTTGCACCTACTGTCCTCATCTTCCCACCAGCTGCTGATCAGGTGGCAACTGAAACAGTCACCATCGTGTGTGTTGCCAATAAATACTTTCCGGATGTTACCGTTACCTGGGAAGTTGATGGCACCACCCAGACCACCGGTATTGAAAATAGCAAAACACCGCAGAATAGCGCAGATTGTACCTATAATCTGAGCAGCACCCTGACCCTGACCAGCACCCAGTATAACAGCCATAAAGAATATACCTGCAAAGTGACCCAGGGTACAACCAGCGTTGTTCAGAGCTTTAATCGTGGTGATTGTGGCGGTGGTGGGAGCGCCCAGCTCAAGAAGAAGCTGCAAGCCCTGAAGAAGAAGAACGCCCAGCTCAAGTGGAAGCTCCAGGCCCTGAAGAAGAAGCTGGCCCAGAAGGGCGGTTCCCATCATCATCACCATCACTAATAA | VL-CL region of rabbit antibody (BAX56586) with leucine zipper and His tag. |
| secM(-AP)–AP | ATGGCCGAACCAAACGCGCCCGCAAAAGCGACAACCCGCAACCACGAGCCTTCAGCCAAAGTTAACTTTGGTCAATTGGCCTTGCTGGAAGCGAACACACGCCGCCCGAATTCGAACTATTCCGTTGATTACTGGCATCAACATGCCATTCGCACGGTAATCCGTCATCTTTCTTTCGCAATGGCACCGCAAACACTGCCCGTTGCTGAAGAATCTTTGCCTCTTCAGGCGCAACATCTTGCATTACTGGATACGCTCAGCGCGCTGCTGACCCAGGAAGGCACGCCGTCTGAAAAGGGTTATCGCATTGATTATGCGCATTTTACCCCACAAGCAAAATTCAGCACGCCCGTCTGGATAAGCCAGGCGCAAGGCATCCGTGCTGGCCCTCACACCACCACCACCACCACTAA | No. 1 in Fig. 3. |
| SKIK–secM(-AP)–AP | ATGTCTAAAATAAAAGCCGAACCAAACGCGCCCGCAAAAGCGACAACCCGCAACCACGAGCCTTCAGCCAAAGTTAACTTTGGTCAATTGGCCTTGCTGGAAGCGAACACACGCCGCCCGAATTCGAACTATTCCGTTGATTACTGGCATCAACATGCCATTCGCACGGTAATCCGTCATCTTTCTTTCGCAATGGCACCGCAAACACTGCCCGTTGCTGAAGAATCTTTGCCTCTTCAGGCGCAACATCTTGCATTACTGGATACGCTCAGCGCGCTGCTGACCCAGGAAGGCACGCCGTCTGAAAAGGGTTATCGCATTGATTATGCGCATTTTACCCCACAAGCAAAATTCAGCACGCCCGTCTGGATAAGCCAGGCGCAAGGCATCCGTGCTGGCCCTCACACCACCACCACCACCACTAA | No. 2 in Fig. 3. |
| secM(-AP) | ATGGCCGAACCAAACGCGCCCGCAAAAGCGACAACCCGCAACCACGAGCCTTCAGCCAAAGTTAACTTTGGTCAATTGGCCTTGCTGGAAGCGAACACACGCCGCCCGAATTCGAACTATTCCGTTGATTACTGGCATCAACATGCCATTCGCACGGTAATCCGTCATCTTTCTTTCGCAATGGCACCGCAAACACTGCCCGTTGCTGAAGAATCTTTGCCTCTTCAGGCGCAACATCTTGCATTACTGGATACGCTCAGCGCGCTGCTGACCCAGGAAGGCACGCCGTCTGAAAAGGGTTATCGCATTGATTATGCGCATTTTACCCCACAAGCAAAACACCACCACCACCACCACTAA | No. 3 in Fig. 3. |
| SKIK–secM(-AP) | ATGTCTAAAATAAAAGCCGAACCAAACGCGCCCGCAAAAGCGACAACCCGCAACCACGAGCCTTCAGCCAAAGTTAACTTTGGTCAATTGGCCTTGCTGGAAGCGAACACACGCCGCCCGAATTCGAACTATTCCGTTGATTACTGGCATCAACATGCCATTCGCACGGTAATCCGTCATCTTTCTTTCGCAATGGCACCGCAAACACTGCCCGTTGCTGAAGAATCTTTGCCTCTTCAGGCGCAACATCTTGCATTACTGGATACGCTCAGCGCGCTGCTGACCCAGGAAGGCACGCCGTCTGAAAAGGGTTATCGCATTGATTATGCGCATTTTACCCCACAAGCAAAACACCACCACCACCACCACTAA | No. 4 in Fig. 3. |
| AP–secM(-AP) | ATGTTCAGCACGCCCGTCTGGATAAGCCAGGCGCAAGGCATCCGTGCTGGCCCTGCCGAACCAAACGCGCCCGCAAAAGCGACAACCCGCAACCACGAGCCTTCAGCCAAAGTTAACTTTGGTCAATTGGCCTTGCTGGAAGCGAACACACGCCGCCCGAATTCGAACTATTCCGTTGATTACTGGCATCAACATGCCATTCGCACGGTAATCCGTCATCTTTCTTTCGCAATGGCACCGCAAACACTGCCCGTTGCTGAAGAATCTTTGCCTCTTCAGGCGCAACATCTTGCATTACTGGATACGCTCAGCGCGCTGCTGACCCAGGAAGGCACGCCGTCTGAAAAGGGTTATCGCATTGATTATGCGCATTTTACCCCACAAGCAAAACACCACCACCACCACCACTAA | No. 5 in Fig. 3. |
| SKIK–AP–secM(-AP) | ATGTCTAAAATAAAATTCAGCACGCCCGTCTGGATAAGCCAGGCGCAAGGCATCCGTGCTGGCCCTGCCGAACCAAACGCGCCCGCAAAAGCGACAACCCGCAACCACGAGCCTTCAGCCAAAGTTAACTTTGGTCAATTGGCCTTGCTGGAAGCGAACACACGCCGCCCGAATTCGAACTATTCCGTTGATTACTGGCATCAACATGCCATTCGCACGGTAATCCGTCATCTTTCTTTCGCAATGGCACCGCAAACACTGCCCGTTGCTGAAGAATCTTTGCCTCTTCAGGCGCAACATCTTGCATTACTGGATACGCTCAGCGCGCTGCTGACCCAGGAAGGCACGCCGTCTGAAAAGGGTTATCGCATTGATTATGCGCATTTTACCCCACAAGCAAAACACCACCACCACCACCACTAA | No. 6 in Fig. 3. |
| secM(-AP)–SKIK–AP | ATGGCCGAACCAAACGCGCCCGCAAAAGCGACAACCCGCAACCACGAGCCTTCAGCCAAAGTTAACTTTGGTCAATTGGCCTTGCTGGAAGCGAACACACGCCGCCCGAATTCGAACTATTCCGTTGATTACTGGCATCAACATGCCATTCGCACGGTAATCCGTCATCTTTCTTTCGCAATGGCACCGCAAACACTGCCCGTTGCTGAAGAATCTTTGCCTCTTCAGGCGCAACATCTTGCATTACTGGATACGCTCAGCGCGCTGCTGACCCAGGAAGGCACGCCGTCTGAAAAGGGTTATCGCATTGATTATGCGCATTTTACCCCACAAGCAAAATCTAAAATAAAATTCAGCACGCCCGTCTGGATAAGCCAGGCGCAAGGCATCCGTGCTGGCCCTCAACGCCTCACCCACCACCACCACCACCACTAA | No. 7 in Fig. 3. |
| secM(-AP)–MSKIK–AP | ATGGCCGAACCAAACGCGCCCGCAAAAGCGACAACCCGCAACCACGAGCCTTCAGCCAAAGTTAACTTTGGTCAATTGGCCTTGCTGGAAGCGAACACACGCCGCCCGAATTCGAACTATTCCGTTGATTACTGGCATCAACATGCCATTCGCACGGTAATCCGTCATCTTTCTTTCGCAATGGCACCGCAAACACTGCCCGTTGCTGAAGAATCTTTGCCTCTTCAGGCGCAACATCTTGCATTACTGGATACGCTCAGCGCGCTGCTGACCCAGGAAGGCACGCCGTCTGAAAAGGGTTATCGCATTGATTATGCGCATTTTACCCCACAAGCAAAAATGTCTAAAATAAAATTCAGCACGCCCGTCTGGATAAGCCAGGCGCAAGGCATCCGTGCTGGCCCTCAACGCCTCACCCACCACCACCACCACCACTAA | No. 8 in Fig. 3. |
| AP-SKIK-SecM(-AP) | ATGTTCAGCACGCCCGTCTGGATAAGCCAGGCGCAAGGCATCCGTGCTGGCCCTTCTAAAATAAAAGCCGAACCAAACGCGCCCGCAAAAGCGACAACCCGCAACCACGAGCCTTCAGCCAAAGTTAACTTTGGTCAATTGGCCTTGCTGGAAGCGAACACACGCCGCCCGAATTCGAACTATTCCGTTGATTACTGGCATCAACATGCCATTCGCACGGTAATCCGTCATCTTTCTTTCGCAATGGCACCGCAAACACTGCCCGTTGCTGAAGAATCTTTGCCTCTTCAGGCGCAACATCTTGCATTACTGGATACGCTCAGCGCGCTGCTGACCCAGGAAGGCACGCCGTCTGAAAAGGGTTATCGCATTGATTATGCGCATTTTACCCCACAAGCAAAACACCACCACCACCACCACTAA | No. 9 in Fig. 3. |
| AP-MSKIK-SecM(-AP) | ATGTTCAGCACGCCCGTCTGGATAAGCCAGGCGCAAGGCATCCGTGCTGGCCCTATGTCTAAAATAAAAGCCGAACCAAACGCGCCCGCAAAAGCGACAACCCGCAACCACGAGCCTTCAGCCAAAGTTAACTTTGGTCAATTGGCCTTGCTGGAAGCGAACACACGCCGCCCGAATTCGAACTATTCCGTTGATTACTGGCATCAACATGCCATTCGCACGGTAATCCGTCATCTTTCTTTCGCAATGGCACCGCAAACACTGCCCGTTGCTGAAGAATCTTTGCCTCTTCAGGCGCAACATCTTGCATTACTGGATACGCTCAGCGCGCTGCTGACCCAGGAAGGCACGCCGTCTGAAAAGGGTTATCGCATTGATTATGCGCATTTTACCCCACAAGCAAAACACCACCACCACCACCACTAA | No. 10 in Fig. 3 |
| sfGFP | ATGGCAAGTAAAGGTGAAGAACTGTTTACCGGCGTGGTTCCGATTCTGGTGGAACTGGATGGTGATGTTAATGGCCATAAATTCAGCGTGCGCGGCGAAGGTGAAGGCGATGCCACCAACGGTAAACTGACGCTGAAATTTATCTGCACCACGGGTAAACTGCCGGTGCCGTGGCCGACCCTGGTTACCACGCTGACGTATGGCGTGCAGTGTTTCAGCCGTTACCCGGATCATATGAAACGCCACGATTTCTTTAAAAGCGCCATGCCGGAAGGTTATGTTCAGGAACGTACCATTTCTTTTAAAGATGATGGCACCTACAAAACGCGCGCAGAAGTGAAATTCGAAGGTGATACCCTGGTTAACCGTATTGAACTGAAAGGCATCGATTTCAAAGAAGATGGTAACATCCTGGGCCATAAACTGGAATACAACTTCAACTCTCACAACGTGTACATCACCGCAGATAAACAGAAAAACGGTATCAAAGCGAACTTCAAAATCCGCCATAATGTGGAAGATGGCAGCGTTCAGCTGGCGGATCACTATCAGCAGAACACCCCGATTGGTGATGGCCCGGTTCTGCTGCCGGATAATCATTACCTGAGCACGCAGTCTGTGCTGAGTAAAGATCCGAACGAAAAACGTGATCATATGGTGCTGCTGGAATTTGTTACCGCGGCCGGTATCACGCACGGCATGGATGAACTGTATAAACTGCATCACCATCACCATCATTAA |  |
| SecM AP-sfGFP with/witout SKIK | ATG[TCTAAAATAAAA]TTCAGCACGCCCGTCTGGATAAGCCAGGCGCAAGGCATCCGTGCTGGCCCTGCCGAACCAGCAAGTAAAGGTGAAGAACTGTTTACCGGCGTGGTTCCGATTCTGGTGGAACTGGATGGTGATGTTAATGGCCATAAATTCAGCGTGCGCGGCGAAGGTGAAGGCGATGCCACCAACGGTAAACTGACGCTGAAATTTATCTGCACCACGGGTAAACTGCCGGTGCCGTGGCCGACCCTGGTTACCACGCTGACGTATGGCGTGCAGTGTTTCAGCCGTTACCCGGATCATATGAAACGCCACGATTTCTTTAAAAGCGCCATGCCGGAAGGTTATGTTCAGGAACGTACCATTTCTTTTAAAGATGATGGCACCTACAAAACGCGCGCAGAAGTGAAATTCGAAGGTGATACCCTGGTTAACCGTATTGAACTGAAAGGCATCGATTTCAAAGAAGATGGTAACATCCTGGGCCATAAACTGGAATACAACTTCAACTCTCACAACGTGTACATCACCGCAGATAAACAGAAAAACGGTATCAAAGCGAACTTCAAAATCCGCCATAATGTGGAAGATGGCAGCGTTCAGCTGGCGGATCACTATCAGCAGAACACCCCGATTGGTGATGGCCCGGTTCTGCTGCCGGATAATCATTACCTGAGCACGCAGTCTGTGCTGAGTAAAGATCCGAACGAAAAACGTGATCATATGGTGCTGCTGGAATTTGTTACCGCGGCCGGTATCACGCACGGCATGGATGAACTGTATAAACTGCATCACCATCACCATCATTAA |  |
| CmlA leader-sfGFP with/without SKIK | ATG[TCTAAAATAAAA]AAGAATGCGGATGCAAGTAAAGGTGAAGAACTGTTTACCGGCGTGGTTCCGATTCTGGTGGAACTGGATGGTGATGTTAATGGCCATAAATTCAGCGTGCGCGGCGAAGGTGAAGGCGATGCCACCAACGGTAAACTGACGCTGAAATTTATCTGCACCACGGGTAAACTGCCGGTGCCGTGGCCGACCCTGGTTACCACGCTGACGTATGGCGTGCAGTGTTTCAGCCGTTACCCGGATCATATGAAACGCCACGATTTCTTTAAAAGCGCCATGCCGGAAGGTTATGTTCAGGAACGTACCATTTCTTTTAAAGATGATGGCACCTACAAAACGCGCGCAGAAGTGAAATTCGAAGGTGATACCCTGGTTAACCGTATTGAACTGAAAGGCATCGATTTCAAAGAAGATGGTAACATCCTGGGCCATAAACTGGAATACAACTTCAACTCTCACAACGTGTACATCACCGCAGATAAACAGAAAAACGGTATCAAAGCGAACTTCAAAATCCGCCATAATGTGGAAGATGGCAGCGTTCAGCTGGCGGATCACTATCAGCAGAACACCCCGATTGGTGATGGCCCGGTTCTGCTGCCGGATAATCATTACCTGAGCACGCAGTCTGTGCTGAGTAAAGATCCGAACGAAAAACGTGATCATATGGTGCTGCTGGAATTTGTTACCGCGGCCGGTATCACGCACGGCATGGATGAACTGTATAAACTGCATCACCATCACCATCATTAA |  |
| WPPP-sfGFP with/without SKIK | ATG[TCTAAAATAAAA]TTCCAGAAGTACGGGATTTGGCCGCCCCCTGCAAGTAAAGGTGAAGAACTGTTTACCGGCGTGGTTCCGATTCTGGTGGAACTGGATGGTGATGTTAATGGCCATAAATTCAGCGTGCGCGGCGAAGGTGAAGGCGATGCCACCAACGGTAAACTGACGCTGAAATTTATCTGCACCACGGGTAAACTGCCGGTGCCGTGGCCGACCCTGGTTACCACGCTGACGTATGGCGTGCAGTGTTTCAGCCGTTACCCGGATCATATGAAACGCCACGATTTCTTTAAAAGCGCCATGCCGGAAGGTTATGTTCAGGAACGTACCATTTCTTTTAAAGATGATGGCACCTACAAAACGCGCGCAGAAGTGAAATTCGAAGGTGATACCCTGGTTAACCGTATTGAACTGAAAGGCATCGATTTCAAAGAAGATGGTAACATCCTGGGCCATAAACTGGAATACAACTTCAACTCTCACAACGTGTACATCACCGCAGATAAACAGAAAAACGGTATCAAAGCGAACTTCAAAATCCGCCATAATGTGGAAGATGGCAGCGTTCAGCTGGCGGATCACTATCAGCAGAACACCCCGATTGGTGATGGCCCGGTTCTGCTGCCGGATAATCATTACCTGAGCACGCAGTCTGTGCTGAGTAAAGATCCGAACGAAAAACGTGATCATATGGTGCTGCTGGAATTTGTTACCGCGGCCGGTATCACGCACGGCATGGATGAACTGTATAAACTGCATCACCATCACCATCATTAA |  |

The coding regions cloned into NdeI site of pET22b are shown. The MSKIK/SKIK tag and His tag are shown in red and light blue, respectively.
